# Cardiac Remodeling and Its Determinants in Anorexia Nervosa Adolescents: Impact of Weight Recovery

**DOI:** 10.3390/children9040458

**Published:** 2022-03-24

**Authors:** Justine Paysal, Jérôme Thireau, Daniel Terral, Emmanuelle Rochette, Philippe Obert, Etienne Merlin, Stéphane Nottin

**Affiliations:** 1 LAPEC UPR 4278, Avignon University, 84000 Avignon, France; jpaysal@chu-clermontferrand.fr (J.P.); philippe.obert@univ-avignon.fr (P.O.); 2 Néonatologie et Réanimation Pédiatrique, CHU Clermont-Ferrand, 63000 Clermont-Ferrand, France; 3 CNRS, INSERM, PhyMedExp, University of Montpellier, 34000 Montpellier, France; jerome.thireau@inserm.fr; 4 Pédiatrie, CHU Clermont-Ferrand, 63000 Clermont-Ferrand, France; dterral@chu-clermontferrand.fr (D.T.); e_rochette@chu-clermontferrand.fr (E.R.); e_merlin@chu-clermontferrand.fr (E.M.); 5 INSERM, CIC 1405, Unité CRECHE, Université Clermont Auvergne, 63000 Clermont-Ferrand, France

**Keywords:** speckle tracking echocardiography, heart rate variability, thyroid hormones, anorexia nervosa

## Abstract

Cardiovascular alterations in anorexia nervosa (AN) adolescents include bradycardia and decreased systolic blood pressure and left ventricular mass. However, their determinants remain poorly understood. We assessed the associations between morphological and functional left ventricular (LV) remodeling, autonomic control by heart rate variability (HRV) analysis, thyroid hormones and brain natriuretic peptide (BNP) levels in AN female adolescents without or with weight recovery (WR). Fifty-nine female adolescents including 16 AN patients without WR (mean age 13.9 years (10–16)), 10 AN patients with WR (15.7 years (12–18)) and 33 controls (14.1 years (10–18)) underwent night heart rate (HR) recording to measure HRV (and especially SD1/SD2, the ratio between instantaneous (SD1) and long-term (SD2) standard deviation of R-R intervals, reflecting sympatho-vagal balance), speckle tracking echocardiography to assess LV global longitudinal strain (GLS) and blood test for dosage of tri-iodothyronine (T3) hormone and NT-proBNP. Compared to controls, AN patients without WR presented with lower HR (55 ± 7 vs. 68 ± 6 bpm; *p* < 0.001), parasympathetic hyperactivity, and higher GLS (−19.2 ± 1.8 vs. −16.9 ± 2.8%; *p* = 0.009). These alterations were partly abolished in AN patients with WR. In a multivariate regression analysis, T3 was the main factor explaining the variance of SD1/SD2, a sympatho-vagal balance marker. NT-proBNP levels were not correlated with cardiac alterations. AN patients had parasympathetic hyperactivity linked with their rate of T3, and a higher GLS. These alterations were partly restored in AN patients with WR.

## 1. Introduction

Anorexia nervosa (AN) is characterized by important weight loss and body composition abnormalities [1]. Most cases are observed in females aged between 12 and 25 years. Cardiovascular complications in AN are frequent; the most common repercussions reported are electrocardiographic abnormalities, hypotension and bradycardia secondary to an increase in parasympathetic tone [2,3]. A cardiac remodeling has also been observed, characterized by a diminished left ventricular (LV) wall thickness and mass [4]. Cardiac output is reduced, mainly due to bradycardia, since left ventricular (LV) stroke volume is unchanged [1]. The diastolic function is marked by a particular ventricular filling profile with decreased A wave [5,6]. A reversibility of these cardiac alterations following weight recovery (WR) has been described [1,2,4,7,8]. Nevertheless, most of the studies used standard ultrasound methods that preclude any conclusion regarding myocardial function in AN patients. Speckle-tracking echocardiography (STE), which allows one to quantify LV strains and which has been shown to have a greater sensitivity for measuring LV performance [9], seems to be an essential tool to complete the study of cardiac function in AN patients.

The underlying mechanisms of these cardiac abnormalities are not clearly understood [10]. It has been suggested that LV morphological changes were linked to food restriction [11], alterations of cardiac afterload [6,12] or biological disturbances (e.g., thyroid hormone levels, growth factors, natremia) [13,14]. The reasons for the parasympathetic hyperactivity are also not fully explained, although an endocrine origin, mediated in particular by thyroid hormones, has been proposed [10,15]. Indeed, it is well-known that thyroid hormones have positive inotropic, dromotropic and chronotropic effects [16] and are decreased in cases of starvation [17,18,19]. Brain natriuretic peptide (BNP) could also be implicated, since it increases in cachexia situations [20,21]) and is able to reduce cardiac mass and loading conditions but also HR [21,22,23]. However, their involvement in AN patients has not been established.

In this context, the aim of our study was to assess LV remodeling and sympatho-vagal balance in AN patients without and with WR, and to explore their potential determinants. We hypothesized that (1) AN patients would exhibit alterations in sympatho-vagal balance, LV morphology and myocardial function compared to controls, (2) these alterations would not be observed in AN patients with WR, and (3) cardiac afterload, thyroid hormones and BNP would be involved in these alterations.

## 2. Materials and Methods

### 2.1. Study Population

The prospective study included female patients with AN who had been diagnosed in a pediatric department of a university hospital in France between March 2019 and January 2020. All patients ranged in age from 10 to 18 years and fulfilled the DSM V criteria for AN (American Psychiatric Association) [24]. The body mass index (BMI) z score was calculated for all participants by the formula established by Cole [25]. AN patients were divided into two sub-groups: AN without or with WR. WR was defined as an increase in BMI of at least 1 z score compared to the patient’s nadir BMI. The mean age of AN patients without WR (*n* = 16) was 13.9 years (10–16), and of AN patients with WR (*n* = 10), 15.7 years (12–18). The control group (*n* = 33) was composed of healthy adolescent girls with normal body mass, free of an eating disorder (no special diet, no bulimic behavior), and without chronic pathology requiring long-term treatment or potentially affecting quality of life. Recruitment was carried out through information posters on the research protocol. Patients and control subjects with chronic disease, congenital heart defects, or positive family history of cardiac disease were excluded. Written informed consent was obtained from the study participants and their guardians. The Ile de France Ethics Committee approved the protocol for this study (18.12.05.66738 CAT 2).

### 2.2. Anthropometrical Data and Body Composition

Body height and body mass were measured. BMI was calculated as body mass.body height^−2^, and body surface area was calculated according to Boyd [26]. Blood pressure was measured using an automatic device (Dynamap PRO 300 V2, General Electric, Boston, MA, USA). Body composition, including body fat mass, lean mass and abdominal fat thickness, was evaluated using a bio-impedance system validated in the measurement of body composition in children [27] (Z-Metrix, BioparHom, Challes Les Eaux, France).

### 2.3. Biological Data

A fasting venous blood sample was taken for biochemical and hormonal determinations, especially NT pro-BNP and TSH, T4, T3, which were measured by a chemiluminescent immunoassay method (Dimension Vista, Siemens Healthcare Diagnostics, Erlangen, Deutchland).

### 2.4. Heart Rate Variability

R–R interval data were recorded by heart rate monitor (V800, Polar Electro Oy, Kempele, Finland) validated to monitor the RR intervals at rest [28] using a chest strap (Polar H10, Polar, Finland). Each R peak due to sinus depolarization was detected, and the normal R–R intervals (NN intervals) were determined. Raw unfiltered R–R data files were exported from the Polar Flow web service as space-delimited .txt files. We used HRV analysis 1.1 to perform analysis with full user control. It allows fast Fourier transform (FFT)-based time-domain analysis and frequency-domain study with beat-by-beat control and review [29].

The recording provided the average resting HR. The time-domain parameters calculated were standard deviation of normal R–R intervals (SDNN, in ms), reflecting total autonomic variability [30,31]; square root of the mean square successive differences between successive normal intervals (RMSSD, in ms), reflecting short-term variations in HR; percentage of normal consecutive R–R intervals differing by >50 ms (pNN50, in %), reflecting cardiac parasympathetic activity; instantaneous standard deviation of R-R intervals (SD1), also reflecting parasympathetic activity; and long-term standard deviation of R–R intervals (SD2) reflecting sympathetic activity [3,31]. The frequency-domain parameters calculated were low-frequency power (LF: 0.04 to 0.15 Hz) for sympathetic activity and high-frequency power (HF: 0.15 to 0.40 Hz) for parasympathetic activity [30,31]. SD1/SD2 and LF/HF ratios, reflecting sympatho-vagal balance, were calculated.

### 2.5. Echocardiographic Evaluation

Echocardiography was carried out with the subject in left lateral decubitus position, with the Vivid Q system (GE Healthcare, Horten, Norway) using a 3.5 MHz transducer (M4S probe). Cine loops were recorded in parasternal short axis, parasternal long axis and apical views and saved for blinded offline analysis (EchoPac, BT 113 version, GE Healthcare, Horten, Norway). All measurements were averaged from five cardiac cycles. STE was performed in accordance with guidelines of the American Society of Echocardiography [32].

LV diameters and myocardial thickness were measured from the parasternal long axis view. LV mass (LVM) was estimated using the Devereux formula and indexed to height^2.7^ (LVM^2.7^), as recommended [33,34]. LV volumes and EF were assessed using the Simpson’s biplane method [32]. LV diastolic function was assessed from peak early (E wave) and atrial (A wave) transmitral flow velocities. TDI velocities (peak E’, A’ and S’) were assessed at the mitral annular level in the different apical views. Peak E’ (recorded on the lateral wall and the septum) and E/E’ ratio were used as indices of LV relaxation and LV filling pressures, respectively [35]. Stroke volume and cardiac output were evaluated from the five-chamber view indexed to the body surface area. Systolic global longitudinal strain (GLS), considered as an index of myocardial systolic function, was calculated from longitudinal strains averaged from the apical 4-chamber, 3-chamber and 2-chamber views [36].

### 2.6. Statistical Analysis

One-way analysis of variance (ANOVA) was used to compare groups, after checking the normality of distribution by Shapiro–Wilk test. In the absence of normal distribution, the nonparametric Kruskal–Wallis test was used. Analyses of covariance (ANCOVA) were used to compare variables that were different between AN patients without and with WR, considering confounding variables such as duration of the disease or changes of BMI z score from nadir BMI. Multiple univariate linear regression analyses were performed between biological, HRV and cardiac ultrasound parameters and blood pressure. Stepwise forward multiple regression analyses were performed to assess which parameters were independent determinants of SD1/SD2, LVM and GLS. All analyses were performed with IBM SPSS software (Version 25.0, IBM Corp., Armonk, NY, USA) and GraphPad Prism (Version 8.0.1).

## 3. Results

**Figure 1:**
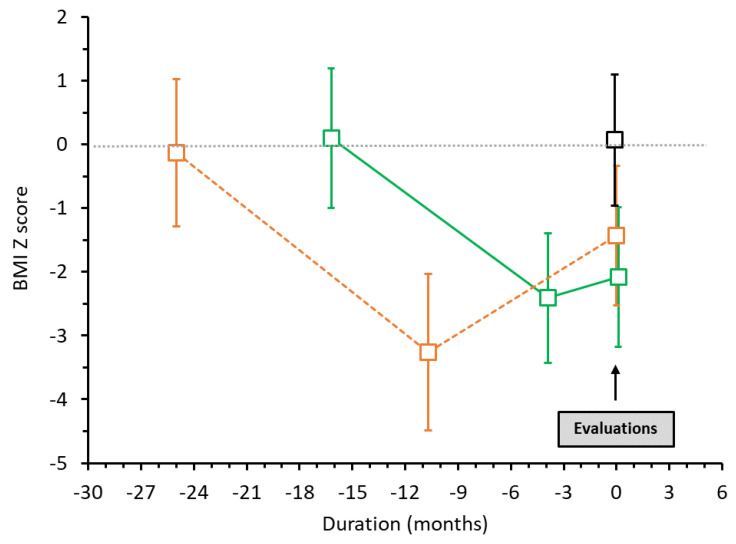
Patterns of the BMI z score from the beginning of the eating disorder in AN patients without WR (in green, solid line) and in AN patients with WR (in orange, dotted line). In black: controls.

The average disease duration was 16 ± 9 and 25 ± 6 months in AN patients without and with WR (*p* = 0.013), respectively. In AN patients with WR, the duration of WR was on average 11 ± 5 months. Figure 1 shows the BMI z score kinetics of our two groups of AN patients from the onset of the disease to our evaluation. BMI z score at the time of assessment was −2.0 ± 1.1 in AN patients without WR and −1.5 ± 1.0 in AN patients with WR (NS), values being in each case significantly different from those in controls (0.1 ± 1.0, *p* < 0.001). AN patients without WR had a mean decrease in their BMI of 3.8% (−0.9 to −7.8%), and AN patients with WR had a mean decrease in their BMI of 4.8% (−2.9 to −7.6%). AN patients without WR had a mean increase in their BMI at time of assessment (compared to their BMI at nadir) of 4.2% (0.0 to 11.1%), and AN patients with WR, of 17.4% (11 to 29.7%).

### 3.1. Clinical Characteristics

Table 1 shows the clinical characteristics and the body composition of AN patients and controls. Patients with AN had significantly lower body mass, BMI and body surface area than controls. BMI was significantly higher in AN patients with than without WR. Percentage of body fat mass was lower, and of body lean mass, higher, in AN patients compared to controls. Percentage of body fat mass was higher in AN with WR compared to AN without WR. AN patients without WR showed significantly lower resting HR and lower SBP than controls. There was no difference in HR between AN patients with WR and controls.

### 3.2. Thyroid Axis and NT-proBNP

**Figure 2:**
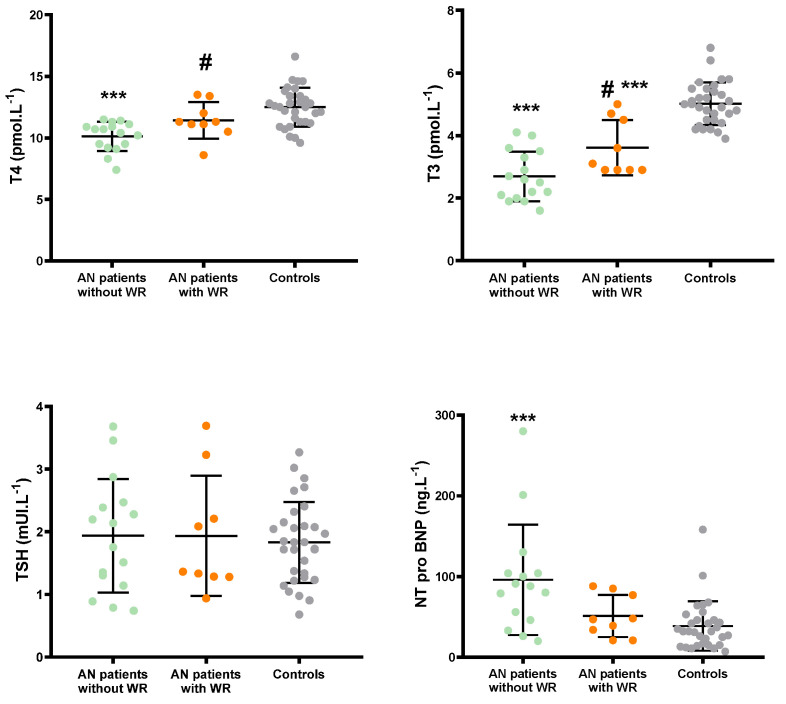
Biological data of AN patients (with, in orange, and without WR, in green) and controls (in grey): T4, T3, TSH and NT-proBNP. ***: significantly different from Controls (*p* < 0.001); ^#^: Significantly different from AN patients with WR (*p* < 0.05).

Evaluations of the thyroid axis and NT-proBNP are presented in Figure 2. Thyroid hormone levels (T3 and T4) were significantly reduced in AN patients without WR compared to AN patients with WR and controls, while no inter-group differences were noticed for TSH. NT-proBNP levels were higher in AN patients without WR than in AN patients with WR and controls.

### 3.3. Sympatho-Vagal Balance

**Figure 3:**
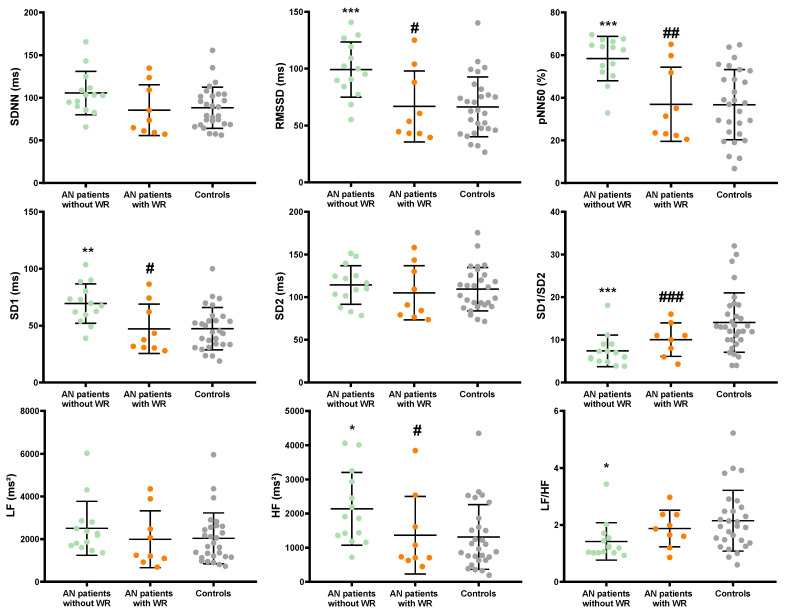
Heart rate variability parameters of AN patients (with, in orange and without WR, in green) and controls (in grey). Significantly different from Controls (*: *p* < 0.05; **: *p* < 0.01; ***: *p* < 0.001); Significantly different from AN patients with WR (^#^: *p* <0.05; ^##^: *p* <0.01; ^###^: *p* < 0.001).

**Figure 4:**
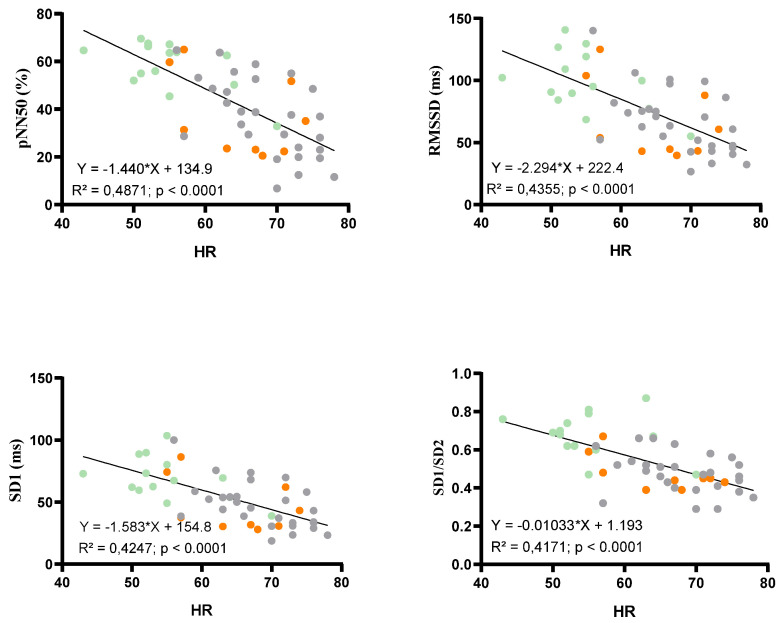
Relations between HR and HRV parameters (PNN50, RMSSD, SD1, SD1/SD2) with a single linear regression. Orange circles: AN patients with WR, green circles: AN patients without WR and grey circles: Controls.

HRV-derived parameters are presented in Figure 3. Time domain HRV parameters reflecting parasympathetic activity (i.e., RMSSD, pNN50, SD1) were significantly increased in AN patients without WR when compared to AN patients with WR and controls. Overall HRV (i.e., SDNN) was higher in AN patients without WR (*p* = 0.077). No difference was found between AN patients and controls for SD2. SD1/SD2 ratio was significantly higher in AN patients without WR than in AN patients with WR and controls. Similar results were observed for frequency domain parameters. HF was greater and LF, reflecting sympathetic activity, was not modified in AN patients without WR compared to AN patients with WR and controls. Significant correlations (Figure 4) were found between HR and pNN50 (R^2^ = 0.49, *p* < 0.0001), RMSSD (R^2^ = 0.44, *p* < 0.0001), SD1 (R^2^ = 0.42, *p* < 0.0001) and SD1/SD2 (R^2^ = 0.42, *p* < 0.0001). When considering the duration of disease as a confounding factor, HR, T3, SD1/SD2 ratio, PNN50, and RMSSD remained significantly different between AN patients without and with WR. However, when considering the variation of BMI z score from nadir BMI as a confounding factor, differences were abolished on HR (*p* = 0.21), T3 (*p* = 0.41), PNN50 (*p* = 0.08), and RMSSD (*p* = 0.28), SD1 (*p* = 0.28), but not on SD1/SD2 (*p* = 0.011).

### 3.4. Left Ventricular Morphology and Function

LV morphology and function are presented in Table 2. LV posterior wall thickness and LVM^2.7^ were lower in AN patients without WR, but differences were abolished in AN patients with WR compared to controls. Both groups of AN patients presented with lower A wave, higher E/A ratio and lower CO index compared to controls. Theses alterations were exacerbated in AN patients without WR. AN patients without WR exhibited higher GLS values when compared to controls, whereas differences were not statistically significant compared to AN patients with WR.

### 3.5. Determinants of Sympatho-Vagal Balance

**Figure 5:**
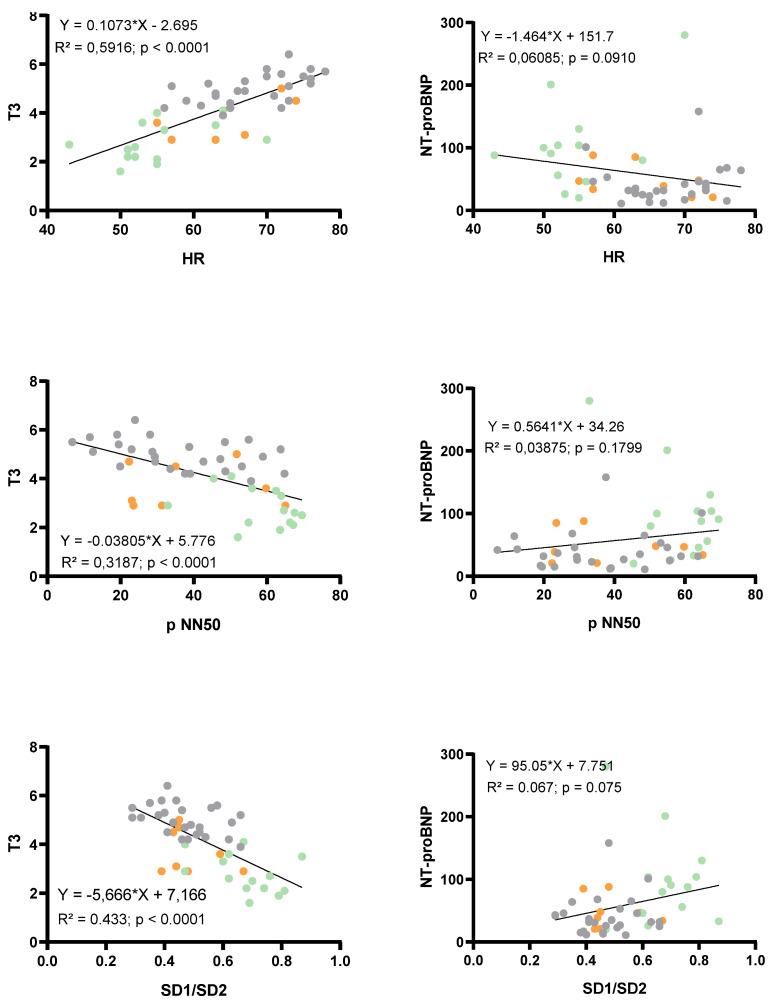
Relations between biological markers (T3 and NT-proBNP) and resting HR or HRV parameters (p NN50, SD1/SD2) with a single linear regression. Orange circles: AN patients with WR, green circles: AN patients without WR and grey circles: Controls.

The most significant correlations were found between T3 and HR (*p* < 0.0001, R^2^ = 0.59), SD1/SD2 (*p* < 0.0001, R^2^ = 0.43), and pNN50 (*p* < 0.0001, R^2^ = 0.32). No significant correlations were found between NT-proBNP and HR, SD1/SD2, or pNN50 (Figure 5). From stepwise forward multivariate regression analyses, only T3 emerged as an independent determinant of SD1/SD2 (*p* < 0.001, R^2^ = 0.42) (Table 3).

### 3.6. Determinants of LVM and GLS

Significant correlations were found between LVM^2.7^ and T3 (*p* = 0.004, R^2^= 0.14), NT-proBNP (*p* = 0.025, R^2^ = 0.09), body fat mass (*p* < 0.0001, R^2^ = 0.27), and SBP (*p* < 0.0001, R^2^ = 0.29). By stepwise forward multivariate regression analyses, body fat mass and SBP were the only independent determinants of LVM^2.7^ (*p* = 0.002, R^2^ = 0.50) (Table 3). Finally, from separate multiple linear regression analyses carried out between biological, LV morphological, HRV data and SBP, no parameters were significantly correlated with GLS, except T3, but the correlation was low (*p* = 0.015, R^2^ = 0.11).

## 4. Discussion

In our study, we observed that (1) parasympathetic activity was higher in AN patients without WR and was closely correlated to their resting bradycardia, (2) cardiac remodeling in these patients was characterized by reduced LV posterior wall thickness and mass but higher GLS, (3) these alterations were not observed in AN patients with WR, and (4) T3 was the only independent determinant of bradycardia and sympatho-vagal imbalance, whereas body fat mass and SBP were the only independent determinants of LVM^2.7^.

### 4.1. Sympatho-Vagal Balance and Resting Bradycardia in AN Patients without or with WR

The first salient findings of the current study were a marked bradycardia as well as sympatho-vagal imbalance due to parasympathetic hyperactivity in AN patients without WR. Indeed, they presented a higher parasympathetic activity, as reflected by the alterations observed in HRV parameters of both temporal and frequency domains (i.e., RMSSD, pNN50, SD1, and HF), but a normal sympathetic activity (i.e., SD2 and LF). Analyses of covariance indicated that the variation of BMI z score from nadir BMI, but not the duration of the disease, mainly explained the differences observed in the parasympathetic activity between groups. A predominance of parasympathetic tone with increased overall variability was also reported in most previous studies in AN individuals [2,10,37,38], but not all [39,40]. Sympathetic modulation was sometimes found reduced [37,38]. The differences observed in the aforementioned studies could be linked to methodological issues [38], the impact of the comorbidities often associated with AN, such as depression and anxiety, or the duration of the disease [38]. Indeed, it does seem likely that there is an evolution from predominantly parasympathetic tone to sympathetic dominant when the disease persists [10,15,37]. The AN patients with WR had a different sympatho-vagal profile, characterized by an absence of parasympathetic hyperactivity and a normal overall variability. Interestingly, their BMI z scores were similar to those of AN patients without WR, strongly suggesting that the sympatho-vagal balance seemed more affected by the WR than by the BMI z score itself.

RMSSD, pNN50, SD1 and the SD1/SD2 ratio were significantly correlated with HR, underlying that parasympathetic hyperactivity in AN patients without WR in large part explained their bradycardia. This parasympathetic hyperactivity may be considered as an adaptive response to caloric deprivation, and the resulting bradycardia as a consequence of the reduction in energy consumption [3]. For that reason, when calorie intake increases, this hyperactivity tends to disappear [2], as shown in our AN patients with WR.

### 4.2. Determinants of Sympatho-Vagal Imbalance and Resting Bradycardia

In our study, we investigated the potential determinants of sympatho-vagal imbalance in AN patients. First, we focused on the levels of thyroid hormones, which are known to be impacted by body mass [17,18,19,41] and affected by the sympatho-vagal balance [16,42,43]. We observed that the levels of T3 and T4 were lower in AN patients without WR than in those with WR or the controls, with no difference in TSH levels, alterations well-known as the “low T3 syndrome”. Moreover, we observed highly significant correlations between thyroid hormones, HR and markers of parasympathetic activity. In multivariate stepwise regression analysis, only T3 emerged as a significant contributor to SD1/SD2, a marker of sympatho-vagal balance. It has been well-described that thyroid hormones have a chronotropic positive effect with increased tissue response to the action of the sympathetic system [16,42,43]. Their implication in AN patients has been debated [6]. Our results strongly supported that T3 could be one major determinant of the sympatho-vagal imbalance and low HR in AN patients without WR. Nevertheless, other potential underlying mechanisms have been suggested, such as electrolyte losses or reduced glycogen content of the myocardium [44].

BNP (or NT-proBNP, inactive form [45]), increased in cachexia situations [46,47] could be responsible for weight loss, while being lipolytic [21,46,47] and reducing hunger [20]. Interestingly, BNP inhibited the sympathetic drive to the heart and enhanced the parasympathetic cardio-cardiac reflex [22], thus resulting in bradycardia. Reciprocally, bradycardia, often accompanied by an increase in stroke volume, could lead to an increase in the level of BNP by ventricular stretch [23]. In this context, we questioned if BNP could be linked to the bradycardia in AN patients. However, we did not observe significant correlations between NT-proBNP levels and HR or HRV-derived parasympathetic parameters. Thus, it seems very unlikely that NT-proBNP levels were involved in resting bradycardia of our AN patients.

### 4.3. Left Ventricular Remodeling in AN Patients without or with WR

In agreement with the literature [2,4], our AN patients without WR had lower LV wall thicknesses and LVM^2.7^. Their cardiac function remained unchanged with normal stroke volume and EF. Only cardiac output was lower, due to bradycardia. The preservation of cardiac function was confirmed by GLS, since it was higher compared to controls. To our knowledge, only one study evaluated GLS in AN patients and found no difference with controls [9].

In AN patients with WR, the differences in LV posterior wall thickness and LVM^2.7^ were abolished compared to controls, strongly suggesting a positive effect of WR on cardiac morphological remodeling. To evaluate the underlying parameters explaining the alteration of LVM^2.7^ in AN, we included, in a multivariate stepwise-forward regression analysis, variables correlated with LVM^2.7^ such as body fat mass, SBP, T3 and NT-proBNP. Despite previous works that observed a link between T3 and cardiac atrophy [15] and a capability of BNP to inhibit cardiac hypertrophy and fibrosis [21,22], our analysis indicated that only body fat mass and SBP were the two main independent predictors of LVM^2.7^. This is perfectly in line with the direct effect of malnutrition causing atrophy, as observed in the skeletal muscle [6] and a decreased afterload from relative hypotension, as previously proposed [6,10,12].

We observed a significant but moderate correlation between T3 and GLS, i.e., lower levels of T3 were associated with higher GLS values. This relationship was surprising if we consider the positive inotropic effect of T3 [16]. One possible explanation is that the low level of T3 did not impact the GLS directly, but indirectly through bradycardia, which in turn generates an increase in the GLS, as suggested by the observation of an inverse correlation between HR and GLS [48]. The clinical significance of the increase in GLS remains poorly understood. It could help maintain normal stroke volume in AN patients despite their lower LVM.

### 4.4. Study Limitations

Our study suffers several limitations. First, the study populations were relatively small. Moreover, we used a cross-sectional design to compare AN patients without and with WR. However, this study design allowed us to obtain a homogeneous group of AN patients with WR (increase in BMI of a least 1 z score). Of note, AN patients with WR had a longer mean duration of illness, but, interestingly, they presented less marked cardiac alterations.

## 5. Conclusions

This study highlighted a number of cardiac consequences of AN, including bradycardia, parasympathetic hyperactivity and LV remodeling characterized by hypotrophy with preserved function. GLS was higher in AN patients. These cardiac alterations appeared reversible, since most differences with controls were abolished in AN patients with WR. Our results supported that the rate of T3 was the main independent determinant of sympatho-vagal balance and bradycardia. Further studies would be helpful to better understand the clinical significance of the increase in GLS in AN patients.

## Figures and Tables

**Table 1:** Baseline characteristics of AN patients without and with WR and controls.

Variables	AN (*n* = 26)		Controls (*n* = 33)
	without WR (*n* = 16)	with WR (*n* = 10)	
Age (years)	13.9 ± 1.6	15.7 ± 1.9 ^#^^,^*	14.1 ± 2.0
Anthropometry			
Height (cm)	159.9 ± 10.3	159.5 ± 7.4	162.6 ± 10.0
Body mass (kg)	39.0 ± 6.9 ***	43.5 ± 9.6 *	51.2 ± 9.8
BMI (kg.m^−2^)	15.2 ± 1.7 ***	16.9 ± 2.2 ^#,^**	19.2 ± 2.3
BSA (m^2^)	1.30 ± 1.15 ***	1.38 ± 0.19 *	1.52 ± 0.19
Bioimpedance analysis			
AFT (mm)	7.4 ± 3.7 **	10.0 ± 3.9	14.1 ± 7.0
Body fat mass (%)	9.8 ± 5.5 ***	15.0 ± 6.1 ^#,^*	20.0 ± 6.6
Lean body mass (%)	86.9 ± 5.9 ***	81.8 ± 5.9 ^#,^*	76.6 ± 6.4
Hemodynamic constants			
Heart rate (bpm)	55 ± 7 ^###,^***	65 ± 7	68 ± 6
Systolic BP (mmHg)	98 ± 16 ***	100 ± 10 ***	110 ± 8
Diastolic BP (mmHg)	65 ± 13	61 ± 7	67 ± 7
Mean BP (mmHg)	76 ± 14 *	74 ± 7 *	81 ± 7

Values are mean ± SD; *: significantly different from controls (*: *p* < 0.05; **: *p* < 0.01; *** *p* < 0.001); ^#^: significantly different between the two subgroups of anorexics (#: *p* < 0.05, ### *p* < 0.001). AN: anorexia nervosa. WR: weight recovery. BMI: body mass index. BSA: body surface area. AFT: abdominal fat thickness. BP: blood pressure.

**Table 2:** Left ventricular morphology and function in AN patients without and with WR and controls.

Variables	AN (*n* = 26)		Controls (*n* = 33)
	without WR (*n* = 16)	with WR (*n* = 10)	
**LV morphology**			
LV septum thickness (cm)	0.71 ± 0.13	0.72 ± 0.19	0.77 ± 0.12
LV posterior wall thickness (cm)	0.63 ± 0.09 **	0.73 ± 0.15 ^#^	0.75 ± 0.12
LV end-diastolic volume (mL)	78 ± 20	81 ± 19	86 ± 18
LV end-systolic volume (mL)	27 ± 8	32 ± 8	31 ± 7
LVM (g)	74 ± 18 **	88 ± 36	96 ± 24
LVM^2.7^ (g.m^−2.7^)	21 ± 5 **	24 ± 7	26 ± 5
**LV function**			
Standard parameters			
E wave (cm.s^−1^)	85 ± 18	82 ± 15	82 ± 14
A wave (cm.s^−1^)	30 ± 7 ***	32 ± 5 **	41 ± 7
E/A	3.0 ± 1.0 ***	2.7 ± 0.7 **	2.0 ± 0.5
Stroke volume index (mL.m^−2^)	38.1 ± 6.4	35.0 ± 4.7	35.6 ± 5.1
CO index (L.min^−1^.m^−2^)	1.9 ± 0.3 ***	2.1 ± 0.5 *	2.5 ± 0.4
Ejection fraction (%)	65 ± 4	61 ± 5	64 ± 6
Tissue Doppler imaging parameters			
E’ (cm.s^−1^)	14.1 ± 1.1	14.6 ± 2.4	15.0 ± 1.5
A’ (cm.s^−1^)	4.8 ± 0.7 ***	5.6 ± 1.0	6.5 ± 1.2
E’/A’	3.0 ± 0.5 ***	2.6 ± 0.4	2.4 ± 0.4
E/E’ _lat_	5.3 ± 1.1	4.8 ± 0.7	4.7 ± 0.8
S’ (cm.s^−1^)	8.1 ± 0.8 **	8.7 ± 1.2	9.1 ± 1.0
Speckle tracking echocardiography parameters			
GLS (%)	−19.1 ± 1.8 **	−18.4 ± 2.3	−16.9 ± 2.8

Values are mean ± SD; *: significantly different from controls (*: *p* < 0.05; **: *p* < 0.01; ***: *p* < 0.001). #: significantly different between the two subgroups of anorexics (^#^: *p* < 0.05). AN: anorexia nervosa. WR: weight recovery. LV: left ventricular. LVM: left ventricular mass. CO: cardiac output. GLS: global longitudinal strain. E’, A’ and S’: Myocardial longitudinal velocity recorded by tissue Doppler imaging during early diastole, late diastole and ejection, respectively.

**Table 3:** Multivariate stepwise analysis.

Model	Variables	B (Standardized Coefficient)	R^2^	*p*
*Relationship between SD1/SD2 and LVM 2.7, body fat mass, T3, NT-proBNP*				
1	T3	−0.651	0.424	<0.001
*Relationship between LVM^2.7^ and body fat mass, EFT, SBP, T3, NT-proBNP*				
1	Body fat mass	0.636	0.404	<0.001
2	Body fat mass Systolic blood pressures	0.443 0.368	0.503	<0.001 0.002

## Data Availability

The datasets used and/or analyzed during the current study are available from the corresponding author upon reasonable request.
